# Hepatocellular uptake index obtained with gadoxetate disodium-enhanced magnetic resonance imaging in the assessment future liver remnant function after major hepatectomy for biliary malignancy

**DOI:** 10.1093/bjsopen/zraa048

**Published:** 2021-07-13

**Authors:** T Notake, A Shimizu, K Kubota, T Ikehara, H Hayashi, K Yasukawa, A Kobayashi, A Yamada, Y Fujinaga, Y Soejima

**Affiliations:** 1 Division of Gastroenterological, Hepato-Biliary-Pancreatic, Transplantation and Paediatric Surgery, Department of Surgery, Shinshu University School of Medicine, Matsumoto, Japan; 2 Department of Radiology, Shinshu University School of Medicine, Matsumoto, Japan

## Abstract

**Background:**

Functional assessment of the future liver remnant (FLR) after major hepatectomy is essential but often difficult in patients with biliary malignancy, owing to obstructive jaundice and portal vein embolization. This study evaluated whether a novel index using gadoxetate disodium-enhanced MRI (EOB-MRI) could predict posthepatectomy liver failure (PHLF) after major hepatectomy for biliary malignancy.

**Methods:**

The remnant hepatocellular uptake index (rHUI) was calculated in patients undergoing EOB-MRI before major hepatectomy for biliary malignancy. Receiver operating characteristic (ROC) curve analyses were used to evaluate the accuracy of rHUI for predicting PHLF grade B or C, according to International Study Group of Liver Surgery criteria. Multivariable logistic regression analyses comprised stepwise selection of parameters, including rHUI and other conventional indices.

**Results:**

This study included 67 patients. The rHUI accurately predicted PHLF (area under the curve (AUC) 0.896). A cut-off value for rHUI of less than 0.410 predicted all patients who developed grade B or C PHLF. In multivariable analysis, only rHUI was an independent risk factor for grade B or C PHLF (odds ratio 2.0 × 10^3^, 95 per cent c.i. 19.6 to 3.8 × 10^7^; *P *<* *0.001). In patients who underwent preoperative portal vein embolization, rHUI accurately predicted PHLF (AUC 0.885), whereas other conventional indices, such as the plasma disappearance rate of indocyanine green of the FLR and FLR volume, did not.

**Conclusion:**

The rHUI is potentially a useful predictor of PHLF after major hepatectomy for biliary malignancy.

## Teaser

Functional assessment of the future liver remnant after major hepatectomy is essential but often difficult in patients with biliary malignancy, because of functional heterogeneity owing to the influence of obstructive jaundice and portal vein embolization. This study investigated whether a novel index using gadoxetate disodium-enhanced MRI (EOB-MRI) could predict posthepatectomy liver failure (PHLF) after major hepatectomy for biliary malignancy. The main findings were that remnant hepatocellular uptake index, a novel quantitative measurement of the functional remnant liver reserve obtained from EOB-MRI, is a useful predictor of PHLF after major hepatectomy for biliary malignancy.

Functional assessment of the future liver remnant after major hepatectomy is essential but often difficult in patients with biliary malignancy, because of functional heterogeneity due to the influence of obstructive jaundice and portal vein embolization. In this study, we investigated whether a novel index using Gd-EOB-DTPA-enhanced MRI (EOB-MRI) could predict post-hepatectomy liver failure (PHLF) after major hepatectomy for biliary malignancy. Our main findings were that rHUI, a novel quantitative measurements of the functional remnant liver reserve obtained from EOB-MRI, is a useful predictor of PHLF after major hepatectomy for biliary malignancy.

Hepatocellular uptake index a promising tool for predicting postoperative liver failure

## Resumen

**Antecedentes:**

La evaluación funcional del remanente hepático futuro (*future liver remnant*, FLR) tras una hepatectomía mayor es esencial, pero muy complicada de establecer en pacientes con neoplasia biliar debido a la ictericia obstructiva y necesidad de embolización portal (*portal vein embolization*, PVE). Este estudio evaluó si un nuevo índice obtenido a través de una resonancia magnética con gadoxetato disódico (EOB-MRI) podría predecir la insuficiencia hepática post-hepatectomía (*posthepatectomy liver failure*, PHLF) después de una hepatectomía mayor por neoplasia biliar.

**Métodos:**

Se calculó el índice de captación hepatocelular del remanente hepático (*remnant hepatocellular uptake index*, rHUI) en pacientes sometidos a EOB-MRI antes de una hepatectomía mayor por neoplasia biliar. Para evaluar la precisión del rHUI en la predicción de la insuficiencia hepática post-hepatectomía de grados B o C (PHLF ≥ B) del *International Study Group of Liver Surgery* se utilizó un análisis de las características operativas del receptor. Se realizó un análisis de regresión logística multivariable en el que se introdujeron diversas variables paso a paso, entre las que se incluía el rHUI y otros índices convencionales.

**Resultados:**

Se incluyeron en este estudio 67 pacientes. El rHUI predijo con precisión la PHLF (área bajo la curva (*area under the curve* (AUC)): 0,896]. Un rHUI < 0,410 predijo todos los pacientes que desarrollaron PHLF ≥ B. En el análisis multivariable, el único factor de riesgo independiente para PHLF ≥ B fue el rHUI (razón de oportunidades, *odds ratio*, OR: 2,0 × 10^3^, i.c. del 95%: 19,6-3,8 × 10^7^, *P* < 0,001). En los pacientes con PVE preoperatoria, el rHUI también predijo con precisión la PHLF (AUC = 0,885), mientras que otros índices convencionales, como la tasa de aclaramiento plasmático del verde de indocianina del FLR (ICGK-F) o el volumen del FLR, no lo hicieron.

**Conclusión:**

El rHUI puede ser un indicador útil de la PHLF después de una hepatectomía mayor por neoplasia biliar.

## Introduction

Posthepatectomy liver failure (PHLF) is a leading cause of life-threatening complications in patients who undergo liver resection^1–6^. In particular, almost all patients with biliary malignancy require major hepatectomy and also have heterogeneous liver parenchyma resulting from preoperative portal vein embolization (PVE) or unilateral biliary drainage. Therefore, accurate measurement of remnant liver function in these patients is essential, and more critical than measurement of total liver function, to avoid PHLF after major hepatectomy.

Various methods, such as CT volumetry and the indocyanine green (ICG) test, have been used for preoperative quantitative assessment of hepatic function^7–10^. Nagino and colleagues^11–13^ reported that the future liver remnant (FLR) plasma clearance rate of ICG (ICGK-F), which is calculated by combining the data from CT volumetry (for FLR volume, FLRV) and ICG clearance tests (for total liver function), was significantly associated with incidence of postoperative mortality. A recent study^13^ revealed that, even with these criteria (ICGK-F below 0.05), the incidence of grade B or C PHLF, as determined by the International Study Group of Liver Surgery (ISGLS), was approximately 20 per cent, and the mortality rate was 1.7 per cent. These data suggest that conventional methods may not be sufficient to accurately estimate remnant functional reserve because they cannot evaluate the heterogeneity of liver parenchyma. Therefore, a more accurate predictor of FLR function is needed.

Gadoxetic acid (Primovist^®^; Bayer-Schering, Berlin, Germany) is a gadolinium-based paramagnetic contrast agent used in MRI of the liver^14^. Gadoxetate disodium is taken up by the hepatocytes via the same transport mechanism as bilirubin. Several studies^15–17^ have reported that gadoxetate disodium-enhanced MRI (EOB-MRI) should provide information for the quantitative evaluation of liver function. Yamada and co-workers^18^ previously reported that the hepatocellular uptake index (HUI) obtained by EOB-MRI facilitated quantitative estimation of liver function and correlated well with the ICG clearance test. The HUI is calculated directly from the FLR, which implies that assessment using this index is more accurate for quantification of FLR function, especially in patients with regional liver heterogeneity.

Several studies^19–22^ have reported that parameters determined from preoperative EOB-MRI could predict PHLF. Remnant HUI (rHUI) is also reported to be a useful predictor of PHLF in patients with hepatocellular carcinoma^23^. However, the main subjects of these studies were patients with colorectal liver metastasis or hepatocellular carcinoma; whether EOB-MRI can usefully predict PHLF in patients with biliary malignancy, in whom regional heterogeneity of the liver is common, remains to be elucidated. The purpose of this study was to evaluate whether rHUI can predict PHLF in patients undergoing major hepatic resection for biliary malignancy.

## Methods

This retrospective study was approved by the Institutional Review Board of Shinshu University (no. 4562), and the requirement for informed consent was waived. The study included patients who underwent major hepatectomy for biliary malignancy between January 2010 and December 2019. Major hepatic resection was defined as a resection involving three or more Couinaud’s segments. Exclusion criteria were: lack of preoperative MRI within 8 weeks, significant cholestasis (bilirubin level over 2.0 mg/dl) at the EOB-MRI examination, and reoperation within 3 days of surgery.

### Preoperative management

Patients with clinical jaundice received preoperative biliary drainage (PBD), with either endoscopic retrograde or percutaneous transhepatic biliary drainage. PBD is usually performed unilaterally on the future remnant hemiliver side^24^.

If scheduled liver resection encompassed more than 60 per cent of the total liver parenchyma, as calculated from serial CT images, preoperative PVE was indicated and scheduled once the serum total bilirubin level had decreased to below 5 mg/dl^25^. The resection was planned 2–3 weeks after PVE, provided that hypertrophy of the FLR (more than 40 per cent of total liver volume) had been confirmed by successive CT scans, and the serum total bilirubin level had dropped to below 2 mg/dl.

### MRI protocol

Preoperative imaging of the entire liver and spleen was performed 20 min after intravenous administration of 0.025 mmol per kg gadoxetate disodium. Single-breath-hold three-dimensional gradient-echo images with fat suppression were obtained (repetition time 3 ms, echo time 1.23 ms, flip angle 14°, slice thickness 3 mm, pixel spacing approximately 1 × 1 mm with various fields of view and image matrices) on a 3.0-T MRI system (Trio Tim, Siemens, Germany) with eight channels in a phased-array body coil. A parallel-imaging technique was used (acceleration factor 2).

### Image analysis

**Fig. 1:**
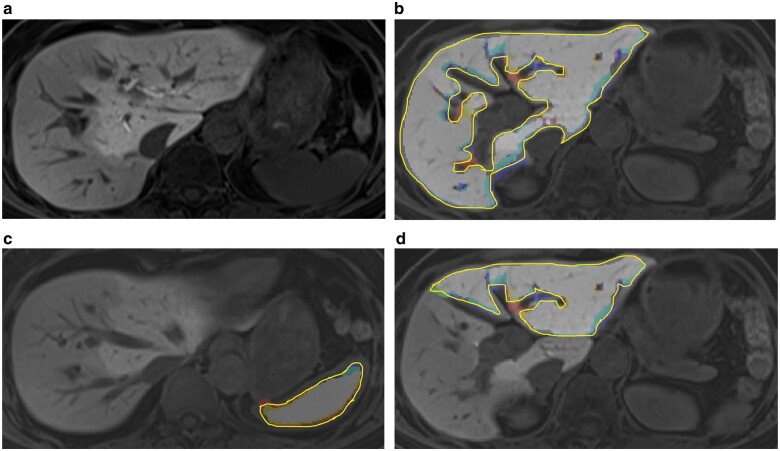
Example of gadoxetate disodium-enhanced MRI and segmentation in a patient with hilar cholangiocarcinoma located in the right hepatic duct with unilateral biliary drainage of the left liver and preoperative portal vein embolization **a** Original gadoxetate disodium-enhanced MRI (EOB-MRI)); **b** segmented whole liver; **c** segmented spleen; and **d** segmented future remnant liver (FLR) after right hemihepatectomy.

Outlines of the FLR and spleen on every slice of preoperative MRI were determined by a board-certified liver surgeon (18 years of experience in liver surgery) and a board-certified radiologist (20 years of experience in diagnostic imaging) with the use of a semiautomatic segmentation tool, developed by MATLAB R2018a (MathWorks, Natick, Massachusetts, USA) (*Fig. 1*). In the procedure to determine the cutting line for hepatectomy, postoperative contrast-enhanced CT or MRI images were used as reference. The volume (rV) and mean signal intensity of the FLR (rL20) and spleen (S20) on preoperative MRI were obtained within the volume included in the outlines. Signal intensities were normalized to maximum signal intensity in all images for each patient. The intraclass correlation coefficient was evaluated for observer concordance of features obtained. From these values obtained by EOB-MRI, rHUI was calculated as: rV×[(rL20/S20) – 1])^18^.

### Estimation of future liver remnant volume

FLR volume (FLRV) was estimated on the basis of Digital Imaging and Communications in Medicine data from CT using three-dimensional volumetry software (Organs Volume Analysis; Hitachi Medical Corporation, Chiba, Japan). The phase in which the liver border was most clearly shown was used in the analyses. The FLR proportion was calculated as the proportion of FLRV to total liver volume. The CT protocol was as follows. The entire liver was imaged on a 64-row CT scanner (Light Speed VCT; GE Healthcare, Little Chalfont, UK). The precontrast phase and nine phases after injecting the intravenous contrast agent were scanned: seven at 6-s intervals, beginning 22 s after injection, and one each at 90 and 210 s after injection. Imaging parameters were: range 25 cm caudal to the upper level of the diaphragm; tube voltage 120 kVp; tube current 300 mA (phases 2–8 and 10) or 500 mA (phases 1 and 9); matrix 512 × 512 pixels; field of view 320 × 320 mm; size of collimation 0.625 mm; reconstruction thickness 2.5 mm. The median effective dose was 48.9 (i.q.r. 48.2–48.9) mSv. A non-ionic iodinated contrast agent (Iopamiron^®^ 370 mg/ml; Bayer Healthcare, Berlin, Germany) was administered intravenously through a 22G catheter in the median cubital vein. The total dose was 100 ml, and the rate of injection 3 ml/s.

### Preoperative and intraoperative data

As preoperative liver function tests, the following serum measurements were recorded: total bilirubin, albumin, aspartate aminotransferase, alanine aminotransferase, prothrombin time, and platelet count. The Child–Pugh grade was calculated and recorded. The preoperative plasma clearance rate of ICG (ICGK) was derived from ICG clearance testing, as described previously^26^. The ICGK-F was calculated as ICGK × FLR proportion^10^. Other intraoperative parameters recorded were duration of operation, blood loss, red blood cell transfusion, and total duration of clamping.

### Definition of posthepatectomy liver failure

PHLF was defined according to the criteria proposed by the ISGLS, comprising increased international normalized ratio (INR) and concomitant hyperbilirubinaemia on day 5 after surgery^27^. The INR cut-off values for prothrombin time and serum bilirubin concentration at this institution were 1.15 and 1.40 mg/dl respectively. Patients with PHLF were further classified into three groups according to severity of liver failure. Grade A PHLF is defined as postoperative impairment of liver function that does not require a change in the patient’s clinical management, which can be accomplished without deviation from the typical postoperative pathway. Grade B PHLF is defined by a deviation from the regular course, but not requiring invasive therapy. The criteria for grade C PHLF include the need for invasive treatment, such as haemodialysis, intubation and mechanical ventilation, extracorporeal liver support, rescue hepatectomy, and transplantation.

### Statistical analysis

Categorical data are presented as numbers with percentages, and continuous variables as median (range). The diagnostic accuracy of MRI parameters for grade B or C PHLF was calculated by means of receiver operating characteristic (ROC) curve analyses, and area under the curve (AUC) was calculated. Multivariable logistic regression analysis was conducted by a stepwise procedure using the minimum Akaike’s information criterion method among the conventional preoperative liver function indicators, including EOB-MRI-related parameters. If data separation occurred in logistic regression analysis, odds ratios and 95 per cent confidence intervals were calculated using Firth’s bias-reduced logistic regression analysis^28^ to evaluate the risk of grade B or C PHLF. Cut-off values for each variable were determined to minimize the Youden index using ROC curve analysis. Odds ratios were calculated to assess associations between cut-off values and occurrence of grade B or C PHLF. *P *<* *0.050 (2-sided) was considered significant. Statistical analyses were performed using JMP^®^ version 14.0 (SAS Institute, Cary, North Carolina, USA).

## Results

**Fig. 2:**
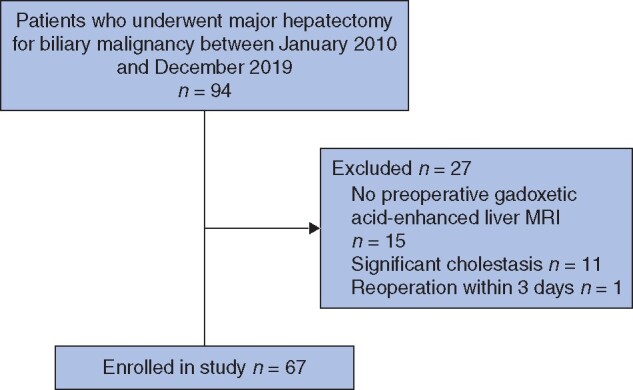
Study flow diagram

Of 94 patients who underwent major hepatectomy for biliary malignancy between January 2010 and December 2019, 67 were included in this study (*Fig. 2*). Clinical and surgical data are summarized in in *Tables 1* and *2*. There were 40 men and 27 women with median age of 71 (range 42–88) years. For preoperative management, PVE was performed in 19 patients (28 per cent), and biliary drainage in 48 (72 per cent). The liver resections were right hemihepatectomy (35, 52 per cent), left hemihepatectomy (27, 40 per cent), right or left trisectionectomy (4, 6 per cent), and central bisectionectomy (1, 1 per cent). Most patients required biliary reconstruction. Pancreatoduodenectomy was performed simultaneously with hepatectomy in 11 patients (16 per cent). The median duration of operation was 657 min, with median blood loss of 610 ml. Blood transfusion was performed in five patients (9 per cent) during the operation.

**Table 1:** Background characteristics of study population according to development of posthepatectomy liver failure

	Overall (*n *=* *67)	Grade B or C PHLF		*P* ^†^
		Yes (*n *=* *8)	No (*n *=* *59)	
**Background data**				
Age (years)*	71 (42–88)	72 (59–81)	71 (42–88)	0·641^‡^
Sex ratio (M : F)	40 : 27	7 : 1	33 : 26	0·087
BMI (kg/m^2^)*	21·9 (15·0–30·7)	19·5 (16·7–24·2)	22·2 (15·0–30.7)	0·012^‡^
Child–Pugh grade				0·119
A	66 (99)	7 (88)	59 (100)	
B	1 (1)	1 (12)	0 (0)	
C	0 (0)	0 (0)	0 (0)	
**Preoperative management**				
Portal vein embolization				0·035
Yes	19 (28)	5 (63)	14 (24)	
No	48 (72)	3 (37)	45 (76)	
Biliary drainage				0·093
Yes	48 (72)	8 (100)	40 (68)	
No	19 (28)	0 (0)	19 (32)	
**Preoperative blood tests** *				
Total bilirubin (mg/dl)	0·70 (0·21–2·15)	0·91 (0·70–2·15)	0·66 (0·21–1·92)	0·003^‡^
AST (units/l)	33 (9–99)	29 (16–57)	33 (9–99)	0·529^‡^
ALT (units/l)	45 (7–227)	40 (17–227)	45 (7–206)	0·504^‡^
Albumin (g/dl)	3·6 (2.8–4·6)	3·4 (2·8–4·1)	3·7 (2·8–4·6)	0·089^‡^
Prothrombin time (%)	90·2 (71·1–130·0)	79·9 (75·6–92·2)	90·6 (71·1–130·0)	0·018^‡^
Platelet count (×10^4^/μl)	22·9 (8·4–48·6)	26·7 (13·6–45·3)	22·7 (8·4–48·6)	0·219^‡^
ICGK*	0·18 (0·10–0·28)	0·15 (0·10–0·22)	0·18 (0·12–0·28)	0.020^‡^
ICGK-F*	0·09 (0·04–0·16)	0·06 (0·04–0·08)	0·09 (0·06–0·16)	< 0·001^‡^
**CT volumetry-related parameters**				
FLRV (ml)*	514 (336–1208)	379 (336–510)	536 (353–1208)	< 0·001^‡^
FLR proportion (%)*	35 (40–87)	38 (37–50)	52 (35–87)	0·004^‡^

Values in parentheses are percentages unless indicated otherwise;

* values are median (range). AST, aspartate aminotransferase; ALT, alanine aminotransferase; ICGK, plasma clearance rate of indocyanine green (ICG); ICGK-F, future liver remnant (FLR) plasma clearance rate of ICG; FLRV, FLR volume.

† Continuous variables were compared using the Wilcoxon rank sum test, while categorical variables were compared using the χ2 test or Fisher’s exact test as appropriate.

‡ Wilcoxon rank sum test.

**Table 2:** Surgical data for study population according to development of posthepatectomy liver failure

	Overall (*n *=* *67)	Grade B or C PHLF		*P* ^†^
		Yes (*n *=* *8)	No (*n *=* *59)	
**Type of hepatectomy**				0·116
Right hepatectomy	35 (52)	7 (88)	28 (47)	
Left hepatectomy	27 (40.5)	0 (0)	27 (46)	
Central hepatectomy	1 (1.5)	0 (0)	1 (2)	
Right trisectionectomy	1 (1.5)	0 (0)	1 (2)	
Left trisectionectomy	3 (4.5)	1 (12)	2 (3)	
**Vascular reconstruction**				0·241
Yes	8 (12)	2 (25)	6 (10)	
No	59 (88)	6 (75)	53 (90)	
**Pancreatoduodenectomy**				0·002
Yes	11 (16)	5 (63)	6 (10)	
No	56 (84)	3 (37)	53 (90)	
**Duration of operation (min)** *	657 (417–1297)	699 (556–1297)	653 (417–1215)	0·155^‡^
**Blood loss (ml)** *	610 (130–4000)	950 (530–2500)	570 (130–4000)	0·039^‡^
**Blood transfusion**				0·104
Yes	5 (7)	2 (25)	3 (5)	
No	62 (93)	6 (75)	56 (95)	
**Total duration of clamping (min)** *	55 (24–139)	61 (36–122)	55 (24–139)	0·818^‡^

Values in parentheses are percentages unless indicated otherwise;

* values are median (range).

† Continuous variables were compared using the Wilcoxon rank sum test, while categorical variables were compared using the χ2 test or Fisher’s exact test as appropriate.

‡ Wilcoxon rank sum test.

Among the 67 enrolled patients, 27 (40 per cent) met the criteria for PHLF: grade A in 19 patients (28 per cent) and grade B or C in eight (12 per cent), including two patients with grade C PHLF. The mortality rate of 3 per cent apply to the whole cohort of 67 patients. Among preoperative factors, total bilirubin (*P *=* *0.003), prothrombin time (*P *=* *0.018), ICGK (*P *=* *0.020), ICGK-F (*P *<* *0.001), and FLRV (*P *<* *0.001) differed significantly between patients with and without grade B or C PHLF (*Table 1*).

### ROC analysis of rHUI for predicting grade B or C PHLF

**Fig. 3:**
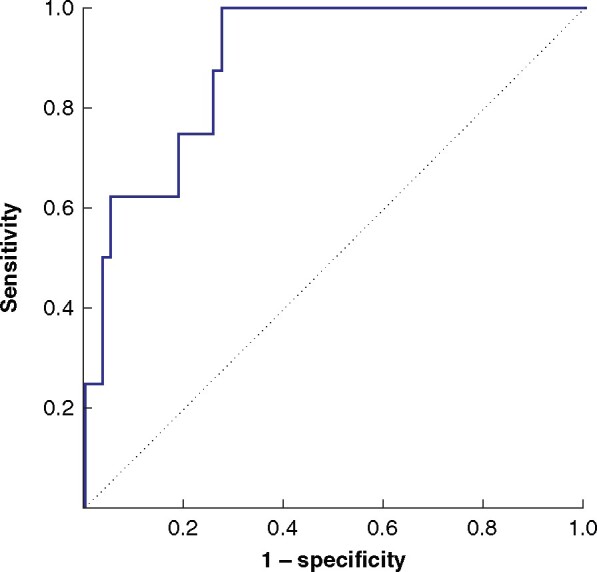
Receiver operating characteristic (ROC) curve for remnant hepatocellular uptake index predicting grade B or C posthepatectomy liver failure Area under the curve 0.896.

**Fig. 4:**
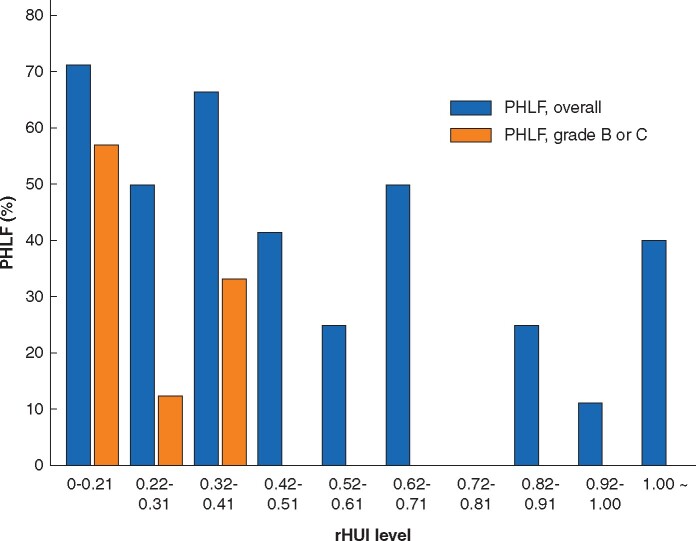
Incidence of posthepatectomy liver failure according to level of remnant hepatocellular uptake index PHLF, posthepatectomy liver failure; rHUI, remnant hepatocellular uptake index.

ROC analyses were done to evaluate the predictive accuracy of rHUI. The AUC was 0.896 (95 per cent c.i. 0.760–0.959; *P *<* *0.001) (*Fig. 3*). The cut-off value of rHUI for predicting grade B or C PHLF was determined as 0.410, with a sensitivity of 1.00 and specificity of 0.73. The incidence of PHLF according to level of rHUI is shown in *Fig. 4*. No patients with an rHUI value exceeding 0.410 developed grade B or C PHLF. Although some patients with an rHUI value greater than 0.410 had PHLF grade A, the incidence of PHLF of all grades was almost inversely proportional to the rHUI value (*r* = –0.743, *P *=* *0.013). Several studies^23^^,^^29^^,^^30^ have reported that the parameters determined from EOB-MRI could predict PHLF in patients who underwent hepatectomy more accurately after adjusting for bodyweight (BW, kg) or body surface area (BSA, m^2^). However, neither BW nor BSA improved the accuracy of rHUI for predicting grade B or C PHLF in the present cohort (AUC for rHUI adjusted for BW 0.595, for rHUI adjusted for BSA 0.622) (*Fig. S1*).

### Accuracy of rHUI compared with conventional parameters for predicting grade B or C PHLF

*Table 3* summarizes the results of ROC analyses for predicting grade B or C PHLF. The AUC for rHUI was larger than those for any other conventional parameters generally used to select appropriate candidates for major hepatic resection.

**Table 3:** Area under the curve for remnant hepatocellular uptake index and other conventional parameters in all patients, and in those who had portal vein embolization

	All patients (*n *=* *67)		PVE (*n *=* *19)	
	AUC	*P*	AUC	*P*
rHUI	0·896 (0·760, 0·959)	< 0·001	0·885 (0·594, 0·976)	0·006
Total bilirubin	0·824 (0·638, 0·910)	0·023	0·921 (0·673, 0·985)	0·004
Serum albumin	0·685 (0·438, 0·858)	0·081	0·657 (0·391, 0·850)	0·381
Prothrombin time (%)	0·757 (0·567, 0·881)	0·012	0·642 (0·316, 0·875)	0·414
ICGK	0·754 (0·502, 0·903)	0·010	0·771 (0·309, 0·962)	0·045
ICGK-F	0·894 (0·724, 0·964)	< 0·001	0·685 (0·265, 0·929)	0·102
FLRV (ml)	0·870 (0·717, 0·947)	< 0·001	0·671 (0·322, 0·897)	0·222
FLR proportion (%)	0·815 (0·650, 0·913)	0·001	0·700 (0·319, 0·920)	0·684

Values in parentheses are 95 per cent confidence intervals. The area under the curve (AUC) for each parameter was calculated from receiver operating characteristic (ROC) curve analyses for predicting grade B or C posthepatectomy liver failure. PVE, portal vein embolization; rHUI, remnant hepatocellular uptake index; ICGK, plasma clearance rate of indocyanine green (ICG); ICGK-F, future liver remnant (FLR) plasma clearance rate of ICG.

*Table 4* shows the results of univariable and multivariable analyses of the rHUI and other preoperative factors as predictors in univariable analysis, rHUI (*P* < 0.001), total bilirubin (*P* = 0.026), prothrombin time (*P* = 0.016), ICGK (*P* = 0.026), ICGK-F (*P* = 0.002), FLRV (*P* < 0.001), and FLR proportion (*P* = 0.001) were associated with development of grade B or C PHLF. These seven factors were included in a stepwise procedure to select appropriate variables for the predictive model. Multivariable stepwise selection analysis identified rHUI below 0.410 as the only factor associated with an increased risk of developing grade B or C PHLF (odds ratio 2.0 × 10^3^, 95 per cent c.i. 19.6 to 3.8 × 10^7^; *P *<* *0.001).

**Table 4:** Univariable and multivariable logistic regression analyses of risk factors for grade B or C posthepatectomy liver failure

	No. of patients	Univariable analysis		Multivariable analysis	
		Odds ratio	*P*	Odds ratio	*P*
**rHUI** *					
< 0·410	18	6·4 × 10^3^ (77·9, 1·1 ×10^8^)	< 0·001	2·0 × 10^3^ (19·6, 3·8 × 10^7^)	< 0·001
≥ 0·410	49	1·00 (reference)		1·00 (reference)	
**Total bilirubin (mg/dl)** *					
> 0·83	20	5·85 (1·08, 31·66)	0·026	34·4 (0·5, 9·7 ×10^3^)	0·070
≤ 0·83	47	1·00 (reference)		1·00 (reference)	
**Serum albumin (g/dl)** *					
< 3·4	14	2·61 (0·54, 12·63)	0·247		
≥ 3·4	53	1·00 (reference)			
**Prothrombin time (%)** *					
< 80	11	7·42 (1·50, 36·60)	0·016	33·2 (0·5, 7·3 × 10^3^)	0·070
≥ 80	56	1·00 (reference)		1·00 (reference)	
**ICGK** *					
< 0·171	26	5·85 (1·08, 31·66)	0·026	60·0 (0·4, 4·8 × 10^4^)	0·081
≥ 0·171	41	1·00 (reference)		1·00 (reference)	
**ICGK-F** *					
< 0·08	26	14·1 (1·69, 128·46)	0·002		
≥ 0·08	41	1·00 (reference)			
**FLR volume (ml)** *					
< 407	16	14·7 (2·58, 83·65)	< 0·001		
≥ 407	51	1·00 (reference)			
**FLR proportion (%)** *					
< 42	25	15·9 (1·82, 139·27)	0·001		
≥ 42	42	1·00 (reference)			

Values in parentheses are 95 per cent confidence intervals.

* Cut-off values were determined to minimize the value of the Youden index. rHUI, remnant hepatocellular uptake index; ICGK, plasma clearance rate of indocyanine green (ICG); ICGK-F, future liver remnant (FLR) plasma clearance rate of ICG.

### Patients with preoperative portal vein embolization

*Table 3* shows the results of the ROC analyses for predicting grade B or C PHLF in 19 patients with preoperative PVE. rHUI accurately predicted grade B or C PHLF (AUC 0.885, *P *=* *0.006) in these patients. On the other hand, conventional parameters, such as ICGK-F (*P *=* *0.102), FLRV (*P *=* *0.222), and FLR proportion (*P *=* *0.684), were less accurate predictors of grade B or C PHLF in patients with preoperative PVE.

**Fig. 5:**
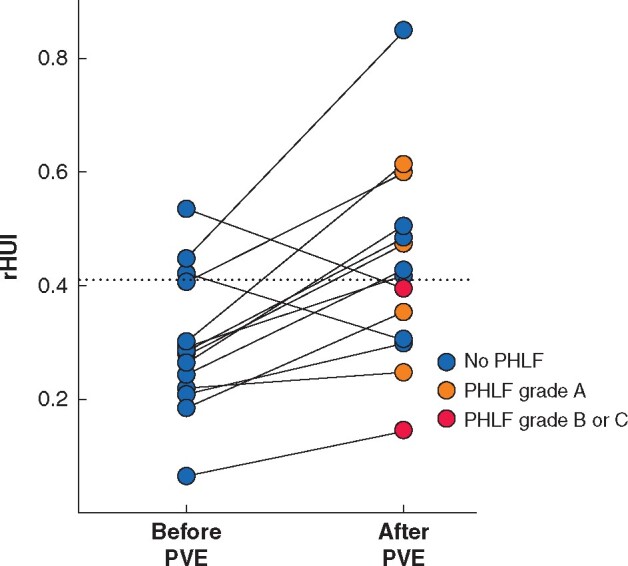
Changes in remnant hepatocellular uptake index after portal vein embolization The dotted line indicates the cut-off value of remnant hepatocellular uptake index (rHUI) for predicting grade B or C posthepatectomy liver failure. PHLF, posthepatectomy liver failure; PVE, portal vein embolization.

Among 19 patients who underwent preoperative PVE, 14 had EOB-MRI both before and after PVE. The median interval between EOB-MRI before and after PVE was 27.5 (14–37) days. Accordingly, rHUI values before and after PVE were compared among these 14 patients. Of 11 patients whose rHUI levels were below the cut-off value of 0.410 before PVE, in seven the rHUI improved to beyond the cut-off value after PVE. No patients whose rHUI level improved beyond the cut-off value developed grade B or C PHLF (*Fig. 5*). Median increases in rHUI and FLRV after PVE compared with before embolization were 1.70 (0.72–2.03) and 1.25 (0.92–1.74) respectively. The improvement in rHUI was significantly better than that of FLRV (*P *=* *0.047). Furthermore, the rHUI/FLRV ratio increased after PVE (1.01×10^−3^ (0.63×10^−3^ to 1.58×10^−3^) *versus* 0.90×10^−3^ (0.45×10^−3^ to 1.45×10^−3^) before PVE), but there was no statistically significant difference (*P *=* *0.080).

## Discussion

The results demonstrate that low rHUI based on EOB-MRI can be a useful predictor of PHLF grade B or higher in patients undergoing major hepatectomy for biliary malignancy. The predictive AUC of rHUI was higher than those of conventional clinical and imaging parameters, such as ICGK-F, FLRV, and FLR proportion. In multivariable analysis, low rHUI was an independent predictor of PHLF.

Both CT volumetry and ICG tests are used for preoperative quantitative assessment of liver function. Additionally, hepatobiliary scintigraphy, such as ^99m^Tc-labelled mebrofenin hepatobiliary scintigraphy^31^ and ^99m^Tc-labelled galactosyl human serum albumin scintigraphy^32^, is reportedly used as an image-based liver function test. Functional liver assessment using EOB-MRI is arguably superior to conventional liver function tests already in clinical use for several reasons. First, EOB-MRI is readily available, and does not require radioactive isotopes. Second, EOB-MRI is already used widely in most centres that specialize in hepatopancreatobiliary surgery, because it has remarkable tumour detection ability owing to its high tissue contrast and high spatial resolution. Third, accurate hepatectomy cutting lines can be planned easily from MRI because of the high resolution of vascular structures, which permits accurate estimation of FLR function. Finally, this novel method can be performed without special tests, such as ICG, which is rarely available in Western countries.

The contrast enhancement effect of gadoxetate disodium in the hepatobiliary phase was due to both uptake by hepatocytes and presence in the extracellular fluid space. Extracellular fluid volumes are reportedly similar in the liver and spleen^33^. Therefore, the contrast enhancement effect should be corrected by the spleen’s signal intensity. Actually, HUI was reported as the factor most significantly correlated with ICGK among various signal intensity-based parameters from EOB-MRI^34^. As parameters comparable to signal intensity-based variables, T1 relaxation time has been reported as an alternative tool for quantifying liver function^34^^,^^35^. In the near future, prediction of PHLF might be further improved using novel MRI techniques, such as T1 relaxation time.

Obstructive cholestasis is often present before surgery in patients with biliary malignancy. EOB-MRI might be affected in patients with obstructive cholestasis, because gadoxetate disodium is taken up by hepatocytes via the same transport mechanism as bilirubin^14^. Eleven patients were excluded from the present study owing to significant cholestasis (total bilirubin level over 2.0 mg/dl). In fact, the rHUI level among these 11 patients with significant cholestasis was significantly lower than that of the study cohort (median 0.360 *versus* 0.521; *P *=* *0.019). These data indicate that concomitant cholestasis might influence gadoxetic acid uptake into hepatocytes, and biliary drainage is essential for accurate analysis of liver function using EOB-MRI.

This study has several limitations. It was a retrospective analysis, with a relatively small sample size and few patients who developed grade B or C PHLF. Further prospective studies with a larger cohort are required. The study included subjects with almost uniform backgrounds, and the cut-off value for rHUI calculated here may not be appropriate for patients with background liver disease. The results may reflect some selection bias, because participants were selected as candidates eligible for hepatectomy using conventional methods for liver function assessment such as CT volumetry and ICG tests. Despite these drawbacks, the authors believe that the results will be of interest to hepatobiliary surgeons because few reports have described the usefulness of EOB-MRI for evaluation of FLR function, especially in patients with liver heterogeneity, such as those who undergo unilateral biliary drainage or PVE.

## Supplementary Material

zraa048_Supplementary_Data
