# Supplementary material for: Hepatocellular uptake index obtained with gadoxetate disodium-enhanced magnetic resonance imaging in the assessment future liver remnant function after major hepatectomy for biliary malignancy
Source: BJS Open. 2021 Jul 13;5(4):zraa048. doi: 10.1093/bjsopen/zraa048 (PMC8275880; doi:10.1093/bjsopen/zraa048)
Supplement: zraa048_Supplementary_Data [file zraa048_supplementary_data.docx]

**Fig. S1 Comparison of ROC curves of independent predictors for predicting PHLF ≥ B**

**
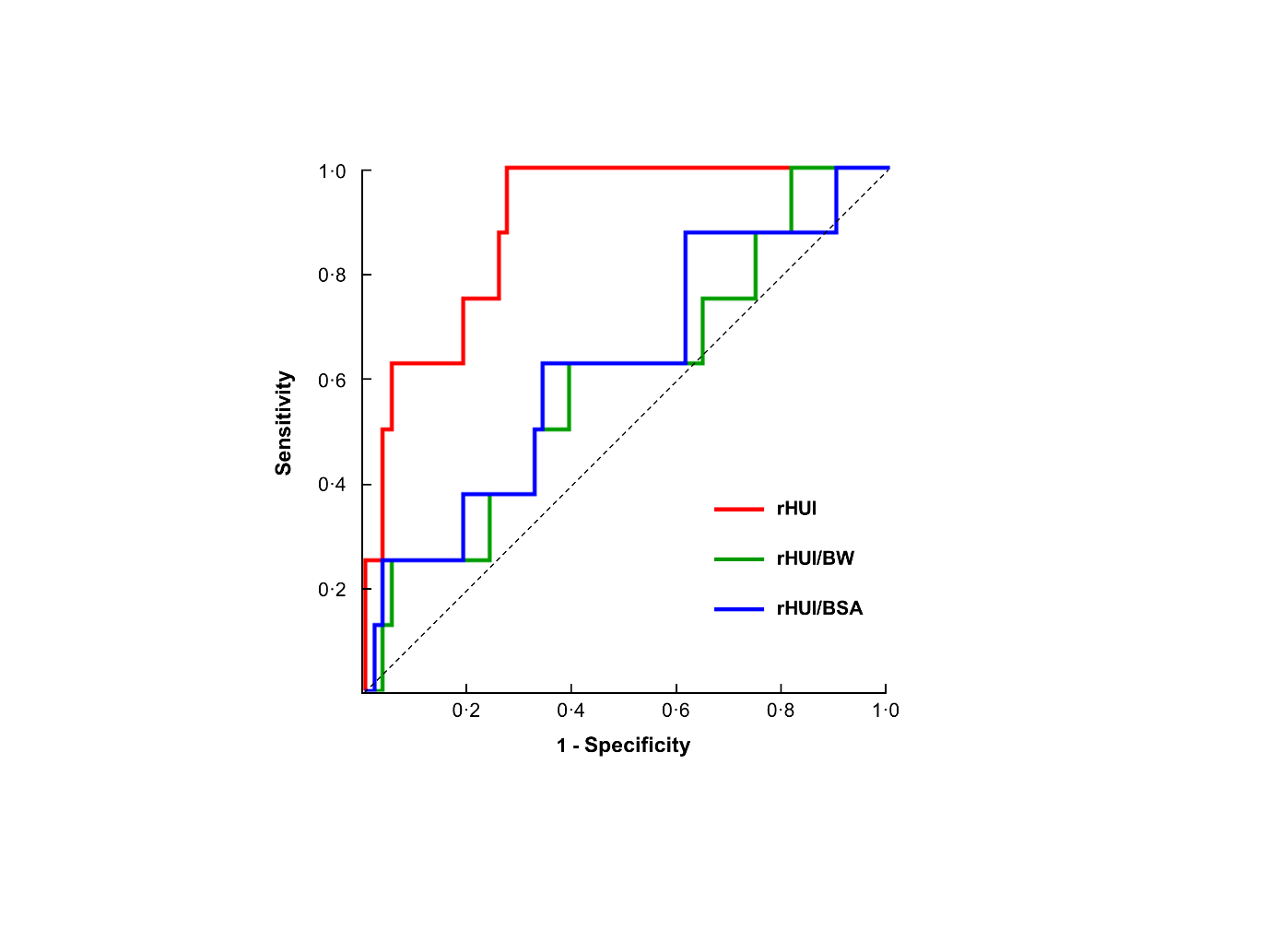
**

rHUI, remnant hepatocellular uptake index; BW, body weight; BSA, body surface area.
